# A white spot syndrome virus microRNA promotes the virus infection by targeting the host STAT

**DOI:** 10.1038/srep18384

**Published:** 2015-12-16

**Authors:** Qian Ren, Ying Huang, Yaodong He, Wen Wang, Xiaobo Zhang

**Affiliations:** 1 Key Laboratory of Animal Virology of Ministry of Agriculture and College of Life Sciences, Zhejiang University, Hangzhou 310058, People’s Republic of China; 2 Jiangsu Key Laboratory for Biodiversity & Biotechnology and Jiangsu Key Laboratory for Aquatic Crustacean Diseases, College of Life Sciences, Nanjing Normal University, Nanjing 210046, China

## Abstract

JAK/STAT pathway plays an important role in invertebrates during virus infection. However the microRNA (miRNA)-mediated regulation of JAK/STAT is not intensively investigated. Viral miRNAs, encoded by virus genome, have emerged as important regulators in the virus-host interactions. In this study, a WSSV (white spot syndrome virus)-encoded miRNA (WSSV-miR-22) was characterized in shrimp during virus infection. The results showed that the viral miRNA could promote WSSV infection in shrimp by targeting the host STAT gene. When the expression of JAK or STAT was knocked down by sequence-specific siRNA, the WSSV copies in shrimp were significantly increased, indicating that the JAK/STAT played positive roles in the antiviral immunity of shrimp. The further findings revealed that TEP1 and TEP2 were the effectors of JAK-STAT signaling pathway. The silencing of TEP1 or TEP2 led to an increase of WSSV copies in shrimp, showing TEP1 and TEP2 were involved in the shrimp immune response against virus infection. Therefore our study presented a novel viral miRNA-mediated JAK/STAT-TEP1/TEP2 signaling pathway in virus infection.

MicroRNAs (miRNAs), 18 to 26 nucleotides (nt) small noncoding RNAs^1^, are initially transcribed by RNA polymerase II or III as long primary RNAs (pri-miRNA), which are further cleaved by Drosha (RNase IIIenzyme) and DGCR8/Pasha (RNA binding protein) into a 60- to 80-nucleotide precursor miRNA (pre-miRNA) with a stem-loop hairpin structure^2^. The pre-miRNAs are then exported to the cytoplasm by exportin-5 and GTP-binding cofactor Ran and further processed by cytoplasm micRNase III enzyme Dicer into approximately 22-nt double-stranded miRNA (miRNA* duplexes)^2^. One strand of the duplex is preferentially selected into the RNA-induced silencing complex (RISC) to generate mature miRNAs^3^. In the complex with an Argonaute (Ago) protein, the mature miRNA binds through its seed sequence (nucleotides 2 to 8) at the 5′ end of an miRNA to a complimentary sequence mostly in the 3′ untranslated region (UTR) of a target mRNA^4^, although there are multiple experimentally validated mRNA targets that lack perfect base-pairing interactions with the miRNA seed region^5^. The relatively short seed sequence of a single miRNA confers the ability to regulate hundreds of mRNAs, as inhibiting their translations or inducing their degradations^6^.

miRNAs, encoded both by cellular and viral genomes, are emerged as important regulators in many biological processes such as proliferation, differentiation, apoptosis, immune response, and tumorigenesis^3^,^7^. Given the importance and versatility of miRNAs, the expression profile of host miRNAs is changed as a result of viral modulation of cellular miRNA expression during virus infection^8^. Host miRNAs can affect viral replication by binding directly to viral mRNAs^9^ or by indirectly modulating host factors to provide a less permissive environment for virus replication^10^. Human miR-122 is an essential component of the biology of hepatitis C virus replication^11^ and therapeutic blocking of miR-122 suppresses hepatitis C viremia in non-human primates^12^. At the same time, the miRNAs encoded by the invading viruses participate in the virus-host interactions by regulating virus or host gene expression to avoid the host defenses and to benefit the viral life cycle^8^. The insect DNA virus *Heliothis virescens* ascovirus (HvAV) encodes a miRNA (virus-encoded miR-1) to down-regulate the expression of the viral DNA polymerase gene^13^. In the marine animal virus white spot syndrome virus (WSSV), the viral miRNAs WSSV-miR-66 and WSSV-miR-68 could target the WSSV genes and further promote WSSV infection^14^. All the viral target genes play negative roles in the WSSV infection in shrimp, indicating that the virus could encode some viral proteins to precisely balance the virus invasion and virus latency in animals^14^.

WSSV is one of the most devastating shrimp pathogens found in farmed penaeid shrimp and other crustaceans, and it has caused serious damage to the world wide shrimp culture industry^15^ Toll, immune deficiency (IMD) and Janus family tyrosine kinase and signal transducer and activator of transcription (JAK/STAT) pathways are regarded as three main signaling pathways regulating humoral immunity of shrimp^16^. Recently, increasing evidences have suggested that JAK/STAT pathway plays an important role in invertebrate organisms during virus infection^17^. In mammals, infectious virus induces the production of interferon and interleukin, which is recognized by cytokine receptors, and then leads to the activation of JAK, which in turn phosphorylates the cytoplasmic domain of the receptor to allow recruitment and phosphorylation of STAT. Activated STAT dimers translocate to the cell nucleus and bind to specific DNA sites, where they act as activators of transcription mechanism^18^. In *Drosophila*, this pathway activates at least two gene families, thioester-containing protein (TEP) and TOT^19^. TEP families of proteins, which are characterized by a unique intrachain β-cysteinyl-γ-glutamyl thioester bond, are classified into two subfamilies: the alpha-2-macroglobulin (A2M) subfamily and the C3 subfamily^20^. Pl-A2M2 from *Pseudomonas libanensis/gessardii* may be important for the immune defense in crayfish intestine and function as a pattern recognition protein in crayfish cuticular tissues^21^.

As reported, WSSV can target host STAT. However, instead of inhibiting or disrupting its activity, WSSV exploits the host STAT by using it to bind to the promoter region of the WSSV immediate early gene *ie1* and thus enhance *ie1* transcription^17^. To better understand the miRNA-mediated regulation of JAK/STAT pathway in shrimp during virus infection, a WSSV-encoded miRNA (WSSV-miR-22) was characterized in the present study. The results indicated that the viral miRNA could promote WSSV infection in shrimp by targeting the host STAT gene.

## Results

### Effects of WSSV-miR22 on virus infection in shrimp

**Figure 1:**
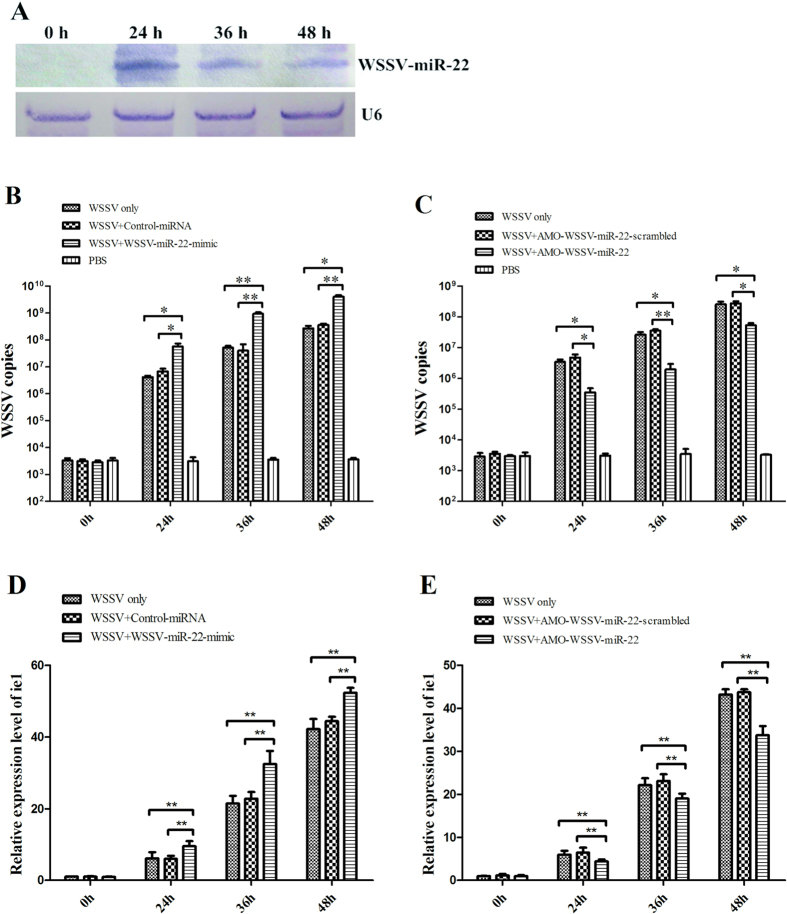
Roles of viral miRNA in virus infection in shrimp *in vivo*. (**A**) The expression profile of WSSV-miR-22 in WSSV-infected shrimp. Northern blotting was conducted using gills of WSSV-infected shrimp at different time points. U6 was used as a control. The numbers indicated the time points post-infection. (**B**) The effect of WSSV-miR-22 overexpression on the virus infection in shrimp. To overexpress the viral miRNA, WSSV-miR-22 and WSSV were co-injected into shrimp. At different time post-infection, the WSSV copies in shrimp were examined. U6 was used as a control. (**C**) The influence of WSSV-miR-22 silencing on the virus infection *in vivo*. AMO-WSSV-miR-22 and WSSV were co-injected into shrimp. At various time post-infection, the WSSV copies in shrimp were evaluated. The numbers showed the time points after WSSV infection in shrimp. (**D**) The affect of WSSV-miR-22 overexpression on the WSSV *ie1* gene expression. WSSV and WSSV-miR-22-mimic or control-miRNA were co-injected into shrimp. At different time points post-infection, the expression of *ie1* was examined with quantitative real-time PCR. (**E**) The impact of WSSV-miR-22 silencing on the *ie1* expression *in vivo*. The *ie1* mRNA level in WSSV-miR-22-silenced shrimp was evaluated with quantitative real-time PCR. In all panels, asterisks indicated significant differences (*p < 0.05; **p < 0.01) between treatments.

In an attempt to reveal the roles of viral miRNA WSSV-miR-22 in virus infection, the expression profile of WSSV-miR-22 was characterized in shrimp *in vivo*. Northern blotting data indicated that WSSV-miR-22 could be detected in WSSV-challenged shrimp at 24, 36 and 48 h post-infection (Fig. 1A). Then WSSV-miR-22 was overexpressed in shrimp. Under the condition that WSSV-miR-22 was overexpressed in shrimp, the number of WSSV copies was examined. As shown in Fig. 1B, the overexpression of WSSV-miR-22 led to significant increases of WSSV copies from 24 h to 48 h post-infection compared with the controls (WSSV-miR-22-scrambled and WSSV only). On the contrary, when the WSSV-miR-22 expression was knocked down by AMO-WSSV-miR-22, the WSSV copies were significantly decreased by comparison with the controls (Fig. 1C). These data indicated that WSSV-miR-22 played a positive role in the virus replication.

To evaluate the effects of WSSV-miR-22 on the expressions of immediate early (ie) genes of WSSV, the *ie1* expression in shrimp treated with WSSV and WSSV-miR-22-mimic or AMO-WSSV-miR-22 was examined. The results showed that the WSSV-miR-22 overexpression led to a significant increase of the *ie1* expression compared with the positive control WSSV only, while the control miRNA had no effect on the expression of *ie1* (Fig. 1D). The WSSV-miR-22 silencing significantly decreased the *ie1* mRNA level (Fig. 1E). The results indicated that WSSV-miR-22 could promote the expression of WSSV immediate early gene.

Taken together, these findings presented that the viral miRNA took a positive effect on the virus infection.

### The interaction between viral miRNA and host *STAT* gene

**Figure 2:**
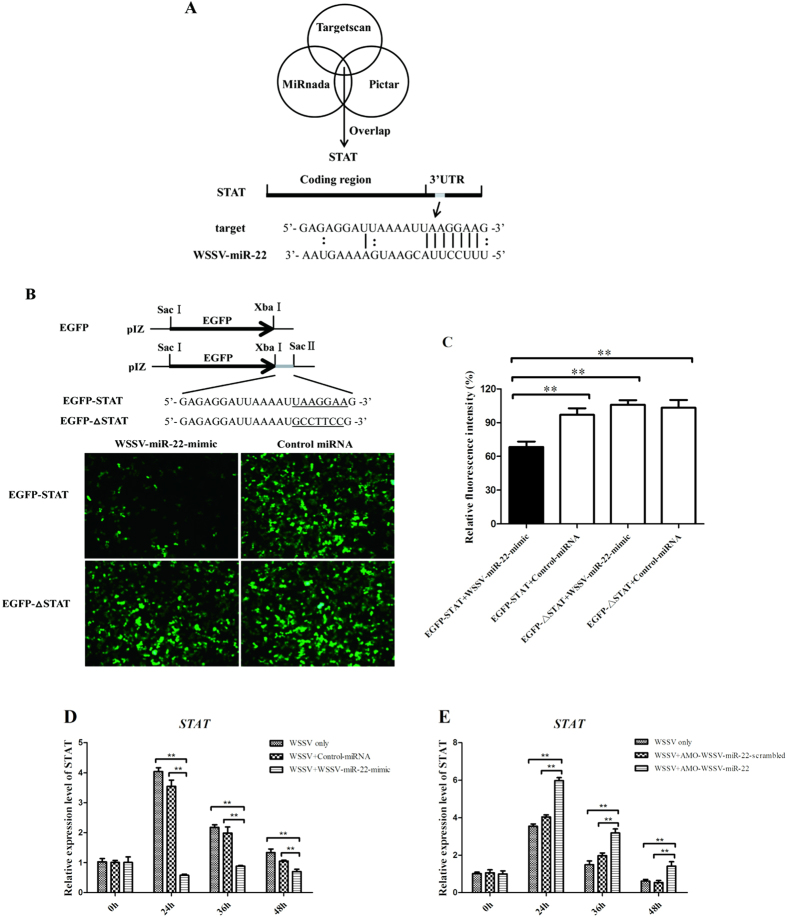
The interaction between viral miRNA and host *STAT* gene. (**A**) The predicted target gene of WSSV-miR-22. The 3′UTR of the *STAT* gene could be targeted by WSSV-miR-22. (**B**) The direct interaction between viral miRNA and host gene. The synthesized WSSV-miR-22 mimic and the plasmid consisting of EGFP and shrimp *STAT* 3′UTR were co-transfected into insect High Five cells. The mutant of *STAT* 3′UTR (EGFP-∆STAT) was included in the co-transfection as a control. At 48 h after cotransfection, the fluorescence of cells was examined. The seed sequence targeted by WSSV-miR-22 was underlined. (**C**) Effects of WSSV-miR22 on the *STAT* gene expression. The relative fluorescence intensity of cells was determined. (**D**) The influence of WSSV-miR-22 overexpression on the expression of shrimp *STAT* gene *in vivo*. WSSV and WSSV-miR-22- mimic were co-injected into shrimp. As a control, WSSV only was included in the injections. At different time post-infection, the expression of *STAT* was examined with quantitative real-time PCR. (**E**) The effect of WSSV-miR-22 silencing on the *STAT* expression *in vivo*. AMO-WSSV-miR-22 was injected into WSSV-infected shrimp to knock down the WSSV-miR-22 expression. WSSV only was used as a control. At different time post-infection, the expression of *STAT* was detected with quantitative real-time PCR. Statistically significant differences between treatments were indicated by asterisks (**p < 0.01).

To reveal the pathways mediated by the viral miRNA, the target genes of WSSV-miR-22 were analyzed. Based on the target prediction using the TargetScan, miRanda, and Pictar algorithms, it was found that WSSV-miR22 could target the shrimp *STAT* gene (Fig. 2A), which is reported to be involved in the antiviral immunity of shrimp^17^, indicating that this viral miRNA might play important roles in virus infection. The BLAST analysis using the non-redundant protein database in GenBank showed that the *Marsupenaeus japonicas* shrimp STAT (GenBank accession number BAI49681.1) shared 96% identity with FcSTAT from *Fenneropenaeus chinensis* (GenBank accession number ACH70130.1), 95% identity with *Penaeus monodon* STAT (GenBank accession number AAQ94739.1) and 84% identity with *Scylla paramamosain* STAT (GenBank accession number AHH29325.1).

To characterize the interaction between the viral miRNA and the host *STAT* gene, the synthesized WSSV-miR-22 and the plasmid EGFP-STAT consisting of EGFP and the STAT 3′UTR were co-transfected into insect High Five cells (Fig. 2B). The results showed that the fluorescence intensity in the co-transfected cells was significantly decreased compared with the intensity in the control cells (Fig. 2B,C). These data presented that WSSV-miR-22 was directly interacted with the *STAT* gene.

In order to explore the interaction between WSSV-miR-22 and *STAT in vivo*, WSSV-miR-22 was overexpressed or silenced in shrimp, followed by the detection of *STAT* mRNA level. The results indicated that the WSSV-miR-22 overexpression led to significantly decreases of the STAT expression compared with the positive control WSSV only, while the control miRNA had no effect on the expression of *STAT*, showing that WSSV-miR-22 inhibited the expression of *STAT* gene in shrimp (Fig. 2D). When the expression of WSSV-miR-22 was knocked down by AMO-WSSV- miR-22, the expression of *STAT* was significantly upregulated (Fig. 2E). These findings showed that WSSV-miR-22 could interact with the host *STAT* gene *in vivo* by targeting its 3′UTR.

### The role of host STAT in virus infection

**Figure 3:**
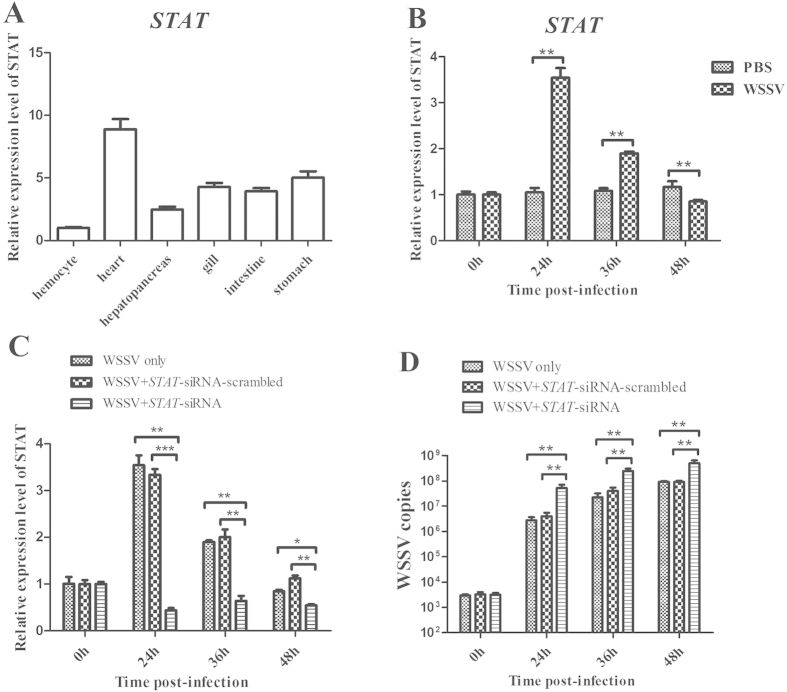
The role of host STAT in virus infection. (**A**) The distribution of *STAT* in various tissues of shrimp. The shrimp *STAT* mRNA levels of hemocyte, heart, hepatopancreas, gill, intestine and stomach were examined using quantitative real-time PCR. Shrimp actin was used as a control. (**B**) The expression profile of *STAT* in shrimp in response to virus infection. Shrimp were infected with WSSV. At different time post-infection, the *STAT* expression in gills was detected with quantitative real-time PCR. (**C**) The silencing of the *STAT* expression in shrimp. The sequence-specific STAT-siRNA was injected into shrimp to knock down the expression of *STAT*. As a control, STAT-siRNA-scrambled was included in the injections. WSSV alone was used as a positive control. (**D**) The influence of STAT silencing on virus infection. The WSSV copies in gills of STAT-siRNA-treated shrimp were quantified using quantitative real-time PCR. In all panels, statistically significant differences between treatments were indicated by asterisks (**p < 0.01).

The data showed that the host *STAT* gene was the target of WSSV-miR-22. Therefore the role of STAT in virus infection was explored in shrimp. Quantitative real-time PCR indicated that the *STAT* mRNA was detected in all the shrimp tissues examined (Fig. 3A). The results revealed that the shrimp STAT was significantly upregulated in response to the WSSV infection (Fig. 3B), showing that the *STAT* gene might play an important role in virus infection.

To evaluate the influence of STAT on virus infection, the *STAT* gene expression was knocked down by sequence-specific siRNA (STAT-siRNA) in shrimp *in vivo*, followed by the detection of virus copy. It was revealed that the expression of *STAT* gene was silenced compared with the control WSSV only, while STAT-siRNA- scrambled had no effect on the STAT gene expression (Fig. 3C), showing that the siRNA was highly specific. Under the condition that the expression of *STAT* gene was knocked down, the number of WSSV copies in shrimp was significantly increased compared with the control (WSSV only) (Fig. 3D). The data indicated that the host *STAT* gene played a negative role in the WSSV infection *in vivo*.

### The influence of host *JAK* gene on virus infection

**Figure 4:**
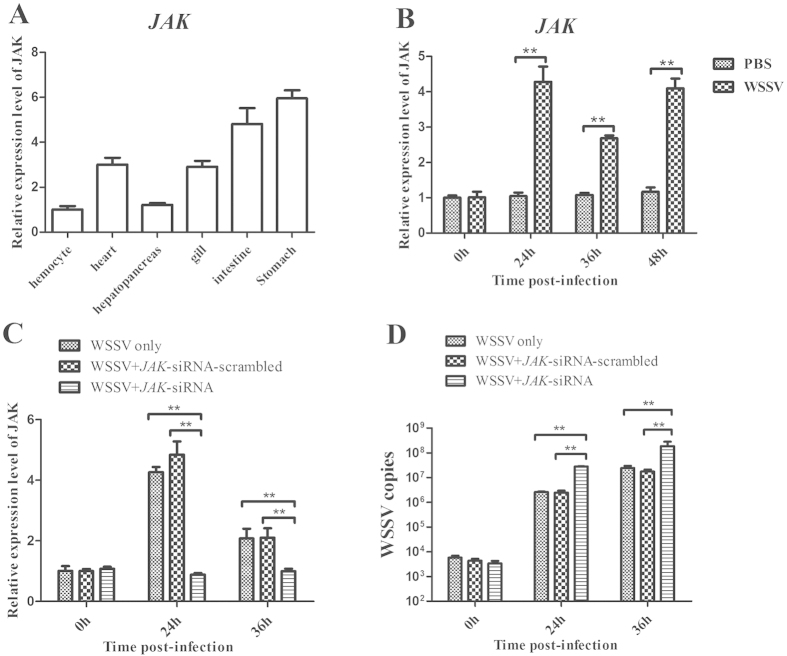
The influence of host *JAK* gene on virus infection. (**A**) The expression profile of *JAK* in shrimp tissues. The mRNA level of *JAK* was determined using quantitative real-time PCR. The shrimp action was used as the control. (**B**) The detection of *JAK* expression in gills of WSSV-challenged shrimp. PBS was used as a control. (**C**) The silencing of *JAK* expression in shrimp. The sequence-specific siRNA (JAK-siRNA) was injected into WSSV-infected shrimp to knock down the expression of *JAK*. At different time post-infection, the JAK expression was examined with quantitative real-time PCR. As a negative control, JAK-siRNA-scrambled was included in the injections. WSSV alone was used as a positive control. (**D**) The effect of JAK silencing on virus infection. The WSSV copies in gills of siRNA-treated shrimp were evaluated using quantitative real-time PCR. In all panels, statistically significant differences between treatments were indicated by asterisks (**p < 0.01).

JAK/STAT signaling pathway has been proved to be very important in antiviral process of vertebrate^22^ and invertebrate^17^,^23^. *JAK*, as one of key components of JAK/STAT pathway, is upstream gene of *STAT*. Therefore the effect of host JAK on virus infection was investigated. As shown in Fig. 4A, the expression of *JAK* was detected in all the examined tissues and it was mainly expressed in the stomach and intestine tissues of shrimp. In response to the WSSV infection, *JAK* was significantly upregulated in shrimp (Fig. 4B). The results suggested that *JAK* was involved in the virus infection in shrimp.

Under the condition that *JAK* gene expression was knocked down by JAK-siRNA in shrimp (Fig. 4C), the number of WSSV copies was evaluated. The data presented that the silencing of *JAK* gene expression led to significant increases of virus copies compared with the control (WSSV only) (Fig. 4D). However, JAK-siRNA-scrambled took no effect on the WSSV infection (Fig. 4D). These findings indicated that JAK played an important role in the virus infection *in vivo*.

### The effects of host TEP1 and TEP2 on virus infection

**Figure 5:**
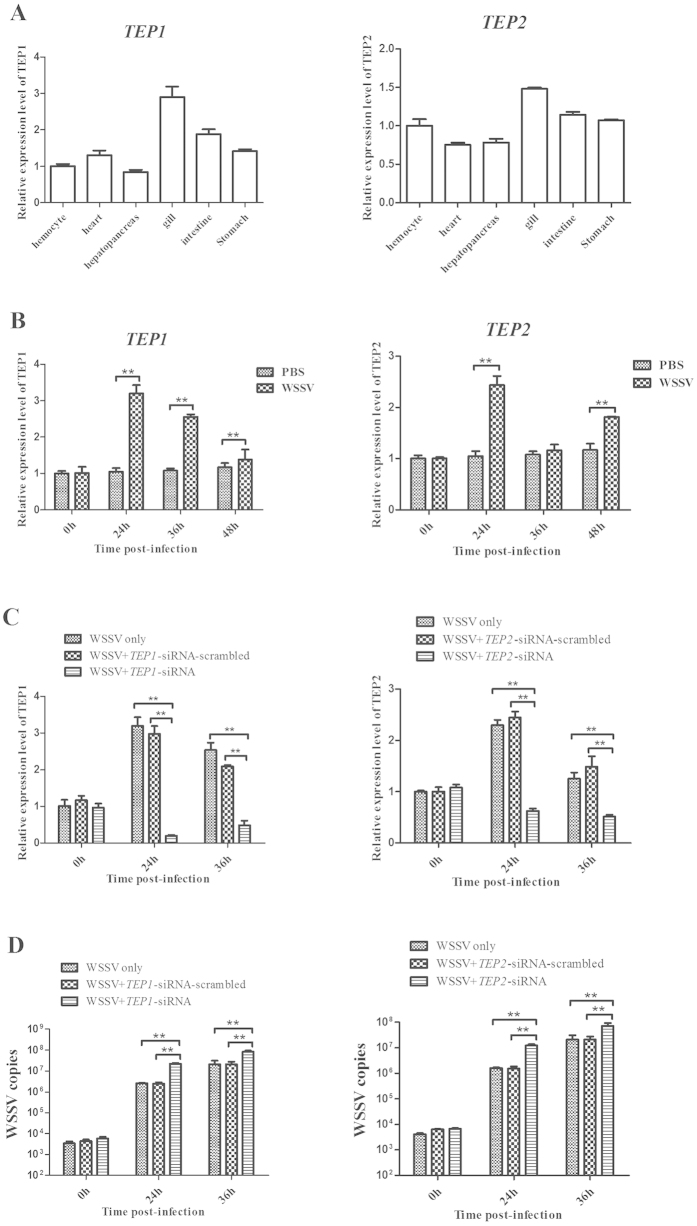
The effects of host TEP1 and TEP2 on virus infection. (**A**) Tissue distributions of *TEP1* and *TEP2* in shrimp. The mRNA levels were determined using quantitative real-time PCR. The shrimp action was used as a control. (**B**) The expression analysis of *TEP1* and *TEP2* in shrimp in response to the virus challenge. Shrimp were infected with WSSV. At different time post-infection, the mRNA levels of *TEP1* and *TEP2* in shrimp were evaluated with quantitative real-time PCR. (**C**) The gene expression silencing of *TEP1* and *TEP2* in shrimp. The sequence-specific TEP1-siRNA or TEP2-siRNA was injected into shrimp. Then the gene expression was examined. As a negative control, siRNA-scrambled was included in the injections. WSSV alone was used as a positive control. (**D**) The detection of WSSV copies in shrimp. The gills of siRNA-treated shrimp were subjected to the quantitative real-time PCR analysis to examine the WSSV copies. WSSV alone was used as a positive control. In all panels, asterisks indicated significant differences between treatments (**p < 0.01).

In *Drosophila melanogaster*, it is reported that the expressions of TEP1 and TEP2 are JAK/STAT-dependent and upregulated in response to the bacteria challenge^21^, suggesting the involvement of TEP1 and TEP2 in the innate immunity of invertebrates. To evaluate the roles of TEP1 and TEP2 in the virus infection, the two genes were characterized in shrimp. The results indicated that both mRNAs of *TEP1* and *TEP2* were detected in all the examined tissues, sharing the similar tissue distributions to those of JAK/STAT (Fig. 5A). In response to the WSSV infection, the expressions of *TEP1* and *TEP2* were significantly upregulated (Fig. 5B), indicating that the two genes played very important roles in the virus infection.

To explore the effects of *TEP1* and *TEP2* on the virus infection, the two genes’ expressions were silenced, followed by the detection of WSSV copies in shrimp. The data presented that the expressions of *TEP1* and *TEP2* were knocked down by sequence-specific siRNAs (Fig. 5C). The results showed that the *TEP1* silencing and the *TEP2* silencing resulted in significant increases of WSSV copies compared with the control (WSSV only), while the TEP1-siRNA-scrambled and TEP2-siRNA- scrambled had no effect on the virus infection (Fig. 5D). These data demonstrated that both *TEP1* and *TEP2* took great effects on the virus infection in shrimp.

### The pathway mediated by viral miRNA in virus infection

**Figure 6:**
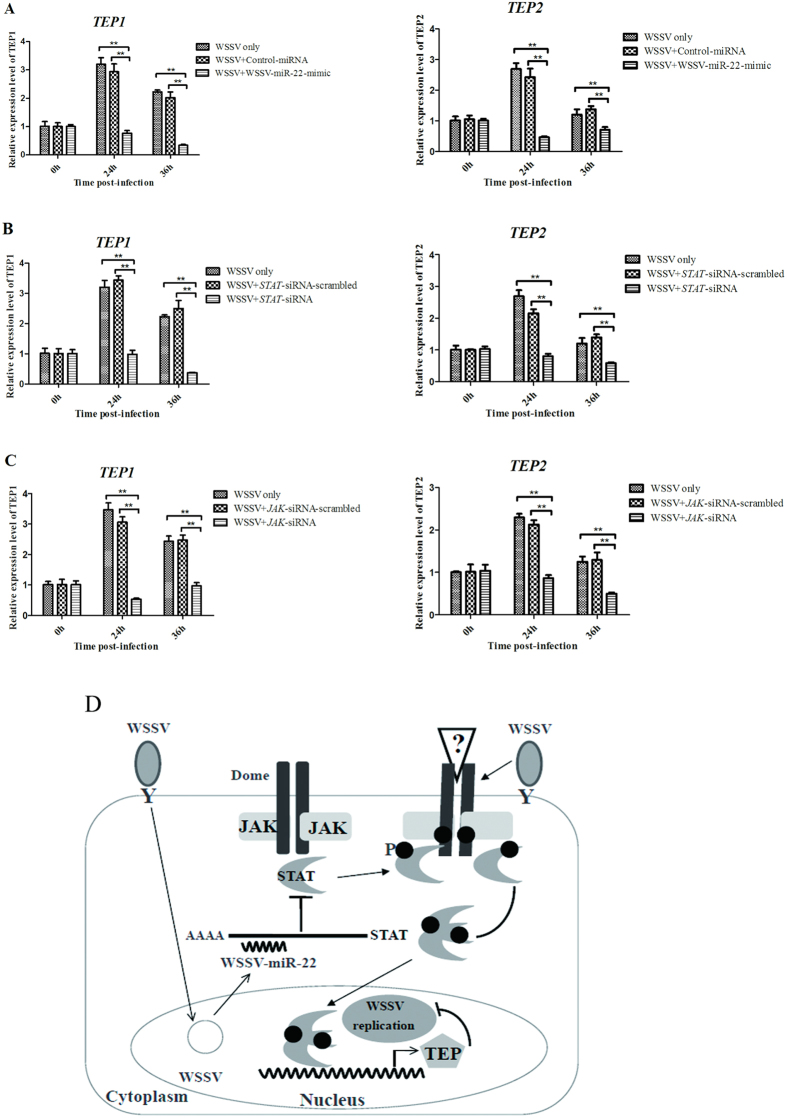
The pathway mediated by viral miRNA in virus infection. (**A**) Effects of viral miRNA silencing on the expressions of *TEP1* and *TEP2*. WSSV-miR-22-mimic and WSSV were co-injected into shrimp. At different time after injection, the mRNA levels of *TEP1* and *TEP2* were quantified by quantitative real-time PCR. (**B**) The relationship between shrimp STAT and TEPs. The WSSV-infected shrimp were injected with STAT-siRNA to silence the expression of *STAT* gene, followed by the quantification of *TEP1* or *TEP2* mRNA using quantitative real-time PCR. (**C**) The relationship between shrimp JAK and TEPs. WSSV and JAK-siRNA were co-injected into shrimp. At different time post-infection, the mRNA levels of *TEP1* and *TEP2* were detected with quantitative real-time PCR. (**D**) Mode for the viral miRNA-mediated pathway in virus infection *in vivo*. Statistically significant differences between treatments were indicated by asterisks (**p < 0.01).

The above data presented that WSSV-miR-22, JAK, STAT, TEP1 and TEP2 were involved in the virus infection in shrimp *in vivo*. Therefore the pathway mediated by the viral miRNA was further explored. The results indicated that the expressions of *TEP1* and *TEP2* were significantly downregulated when the expression of WSSV-miR-22 was overexpressed in shrimp (Fig. 6A), suggesting that the viral miRNA, TEP1 and TEP2 shared the same pathway.

To reveal the relationship between STAT and TEPs (TEP1 and TEP2), the expression of *STAT* was knocked down by STAT-siRNA in shrimp. It was found that the *STAT* silencing led to significant decreases of both *TEP1* and *TEP2* mRNA levels compared with the controls (Fig. 6B), showing that *TEP1* and *TEP2* were the downstream genes of *STAT*. To evaluate the effect of *JAK* expression silencing on the expressions of *TEP1* and *TEP2*, the JAK-siRNA-treated shrimp, which were simultaneously infected with WSSV, were subjected to the detections of *TEP1* and *TEP2* mRNA levels. The quantitative real-time PCR data presented that both the *TEP1* and *TEP2* were downregulated in the JAK-silenced shrimp by comparison with the controls (Fig. 6C).

Taken together, these findings revealed that the viral miRNA (WSSV-miR-22) could inhibit the JAK/STAT-TEP1/TEP2 signaling pathway by targeting the host *STAT* gene, leading to the virus infection in shrimp (Fig. 6D).

## Discussion

The activation/inactivation of transcription factors affect the expressions of a large number of genes, leading to the changes of biological processes. During the virus-host interactions, the regulation of transcription factors’ expression becomes the key issues. The host’s transcription factors, such as STAT, are often selected by virus as targeted sites. Through the protein-protein interactions, the host’s transcription factors can be utilized by virus to enhance its infection. It is evidenced that STAT, an important transcription factor, is involved in the course of virus infection. The measles virus (MV) phosphoprotein (P) can interact with the linker domain of STAT1 and subsequently inhibit the JAK/STAT activation^24^. Hepatitis C virus (HCV) core protein is required for the production of infectious viruses through the interaction with the JAK protein^25^. On the basis of protein-protein interactions, the activity of transcription factor can be turned off by virus. However, this turnoff of transcription factor activity may result in disorders of many biological processes. In recent years, it is found that miRNAs, a kind of regulators participating in the post-transcription regulations of large number of protein-encoding genes, can regulate the genes’ expressions by fine tuning^26^. In this context, miRNA-mediated expression regulation of transcription factor may be a better strategy for the virus-host interactions. To reveal the mechanisms of virus-host interactions, the host miRNAs involved in the regulation of transcription factor expression have attracted more and more interests. In human, the host miR-146 and miR-21 are used by human immunodeficiency virus (HIV) and HCV to downregulate the expressions of IRAK1 and TRAF6, leading to the decrease of the activity of NF-κB^27^,^28^. The upregulation of human miR-373 by the HCV infection can target the JAK gene and then impair STAT phosphorylation and inhibit the JAK/STAT signal pathway^29^. Up to date, however, the regulation of transcription factor expression mediated by the virus miRNAs has not been explored. In this study, the results indicated that the viral miRNA could promote the WSSV infection by targeting the shrimp STAT. Our study revealed a novel viral miRNA-mediated JAK/STAT-TEP1/TEP2 signaling pathway in the virus-host interactions.

At present, it is reported that WSSV can encode more than 80 viral miRNAs^30^,^31^. Among these viral miRNAs, WSSV-miR-N24 targets shrimp caspase 8 gene, leading to the inhibition of apoptotic activity and the promotion of virus infection^32^. Both WSSV-miR-66 and WSSV-miR-68 can enhance virus replication by inhibiting the virus genes’ expression^14^. In the present study, the findings contributed a novel aspect of viral miRNA in the virus infection by targeting the host’s transcription factor gene. As well known, the JAK/STAT signaling pathway is highly conserved from vertebrates to invertebrates and plays an important role in the antiviral immune response^29^,^33^. The activation of STAT, the transcription factor of JAK/STAT signaling pathway, triggers expressions of the effect genes. However, the effect genes regulated by the JAK/STAT pathway have not extensively characterized. In the present investigation, it was indicated that the expressions of TEP1 and TEP2 were regulated by JAK/STAT. TEPs have three different families, including alpha-2-macroglobulins (A2Ms), C3/C4/C5 complement factors, and insect TEPs (iTEPs)^34^. Macroglobulin complement-related factors, which belong to the iTEP family, are crucial effectors to defense the flaviviral infection of *Aedes aegypti*^35^. Our study revealed that the shrimp TEP1 and TEP2, the effectors of JAK-STAT signaling pathway, played important roles in the virus-host interactions. In this context, the regulation of transcription factor’s expression mediated by viral miRNA might represent a key issue in the virus-host interactions.

## Materials and Methods

### Shrimp culture and WSSV challenge

The *Marsupenaeus japonicas* shrimp (approximately 10 g in body weight and 10 to 12 cm in length) were purchased from an aquaculture market in Hangzhou, Zhejiang Province, China. Before treatments, the shrimp were cultured in groups of 20 individuals in laboratory tanks containing 80 liters of aerated seawater at room temperature. To ensure that the shrimp were WSSV-free before experiments, PCR using WSSV-specific primers (5′-TATTGTCTC TCCTGACGTAC-3′ and 5′-CACATTCTTCACGAGTCTAC-3′) were conducted. Then the virus-free shrimp were infected with 100 μl of WSSV virus solution at 10^5^ copies/ml by intramuscular injection into the lateral area of the fourth abdominal segment. At different times post-infection (0, 24, 36, and 48 h), five shrimp were randomly collected for each treatment and stored for later use. In the following assays, the shrimp gills were employed. As well known, the shrimp gill is one of the immune organs and is an important target organ of WSSV infection. On the other hand, the shrimp *JAK*, *STAT*, *Tep1* and *Tep2* genes are highly expressed in shrimp gills.

### The detection of miRNA by Northern blotting

Total RNAs were extracted from shrimp gills by using the mirVanaP^TMP^ miRNA isolation kit (Ambion, USA) according to the manufacturer’s protocol. After separation on a denaturing 15% polyacrylamide gel containing 8 M urea, the small RNAs were transferred to a Hybond-N+ membrane (Amersham Biosciences, Buckinghamshire, United Kingdom). Subsequently, the RNAs were cross-linked under UV light (Ultra-Violet Products Ltd., USA). The membrane was prehybridized in DIG Easy Hyb Granules buffer (Roche, Basel, Switzerland) for 30 min and then hybridized with digoxigenin (DIG)-labeled probes completely complementary to WSSV-miR-22 (5′-UUUCCUUACGAAUGAAAAGUAA-3′) at 42 °C overnight. The DIG-labeled U6 probe (5′-GGGCCATGCTAATCTTCTCTGTATCGTT-3′) was used as a control. Immunological detection was performed using the DIG High Prime DNA labeling and detection starter kit II (Roche, Basel, Switzerland).

### The silencing or overexpression of WSSV-miR-22 in shrimp

Based on the sequence of WSSV-miR-22 (5′-UUUCCUUACGAAUGAAAAG UAA-3′), the WSSV-miR-22 was synthesized with *in vitro* transcription T7 kit for miRNA synthesis (Takara, Japan). The sequence of WSSV-miR-22 was scrambled to generate the control WSSV-miR-22-scrambled (5′-GCGAUAAAUAGUAAUACUUAUC-3′). The synthesized miRNA was dissolved in miRNA solution (50 Mm Tris-HCl, 100m MNaCl, pH 7.5) and quantified by spectrophotometry. To overexpress the viral miRNA, the miRNA (15 μg) and WSSV (10^5^ copies/ml) were co-injected into virus-free shrimp at a volume of 100 μl per shrimp. At 16 h after the co-injection, the miRNA (15 μg) (100 μl /shrimp) was injected into the same shrimp. As controls, WSSV-miR-22-scrambled, WSSV alone (10^5^ copies/ml) and physiological saline (0.85% NaCl) were included in the injections.

To knock down the WSSV-miR-22 expression, an anti-miRNA oligonucleotide (AMO) was injected into WSSV-infected shrimp. AMO-WSSV-miR-22 (5′- TTACTTTTCATTCGTAAGGAAA-3′) was synthesized (Sangon Biotech, Shanghai, China) with a phosphorothioate backbone and a 2′-O-methyl modification at the 6th, 18th and 20th nucleotides. AMO (10 nM) and WSSV (10^5^ copies/ml) were co-injected into virus-free shrimp at a volume of 100 μl per shrimp. At 16 h after the co-injection, AMO (10 nM) (100 μl /shrimp) was injected into the same shrimp. As controls, AMO-WSSV-miR-22-scrambled (5′-ATGTAGATTCAATTTGAACCTT-3′), WSSV alone (10^5^ copies/ml) or physiological saline (0.85% NaCl) was injected into shrimp.

For each treatment, 20 shrimp were used. At different times post-infection (0, 24, 36 and 48 h), five shrimp were randomly collected for each treatment and subjected to subsequent analysis. All the experiments were biologically repeated three times.

### The quantitative real-time PCR analysis of WSSV copies

Quantitative real-time PCR was performed to examine the WSSV copies in gills of WSSV-infected shrimp. The viral DNA was extracted from shrimp gills using the SQ tissue DNA kit (Omega-Bio-Tek, USA), and then the WSSV copies were detected by real-time PCR with WSSV-specific primers (5′-TTGGTTTCAGCCCGAGATT-3′ and 5′-CCTTGGTCAGCCCCTTGA-3′) and WSSV-specific TaqMan probe (5′-FAM-TGCTGCCGTCTCCAA-TAMRA-3′). The 25 μl PCR solutions contained 12.5 μl of Premix ExTaq (TaKaRa, Japan), 0.5 μl of 10 μM forward primer, 0.5 μl of 10 μM reverse primer, 1 μl of 10 μM TaqMan fluorogenic probe, 1 μl of DNA template, and 9.5 μl distilled water. The predenaturation stage of the PCR program was 95 °C for 1 min, followed by the amplification stage consisting of 40 cycles of 95 °C for 30 s, 52 °C of 30 s, and 72 °C for 30 s.

### The prediction of target genes

To predict the target genes of viral miRNA, the shrimp genome sequence was employed with three independent computational algorithms TargetScan 5.1 (http://www.targetscan.org), miRanda (http://www.microrna.org/) and Pictar (http://pictar.mdc-berlin.de/).

### Plasmid construction

To characterize the direct interaction between WSSV-miR-22 and the shrimp *STAT* gene, the 3′UTR of *STAT* and enhanced green fluorescent protein (EGFP) gene were cloned into a pIZ/EGFP V5-His vector (Invitrogen, USA). The EGFP gene was amplified from the pEGFP vector (BD Biosciences, USA) using EGFP-specific primers (5′-AAGAGCTCGGATCCCCGGGTA-3′ and 5′-AATCTAGAGTCGCGGCCGCTTTA-3′). Subsequently the *STAT* 3′UTR was cloned into the pIZ vector downstream of EGFP with sequence-specific primers (5′-CGAGCTCACCATGGGGTCGTTGTGGAACAGAGCAC-3′ and 5′-GCTCTAG ACTCAAATGCCGGTGAACATATTTCC-3′). As a control, the *STAT* 3′UTR sequence (TAAGGAA) complementary to the WSSV-miR-22 seed sequence was mutated to GCCTTCC, yielding the EGFP-∆STAT construct. All the recombinant plasmids were confirmed by sequencing.

### Cell culture, transfection, and fluorescence assays

Insect High Five cells (Invitrogen, USA) were cultured at 28 °C in Express Five serum-free medium (SFM) (Invitrogen) containing l-glutamine (Invitrogen). When the cells were at about 70% confluence, they were transfected with 6 μg of EGFP, EGFP-STAT or EGFP-∆STAT. At the same time, the cells were transfected with 300 nM of either synthesized WSSV-miR-22 or a synthesized control miRNA. All the miRNAs were synthesized by Shanghai Gene Pharma Co., Ltd. (Shanghai, China). The transfections were carried out in triplicate with Cellfectin transfection reagent (Invitrogen) according to the manufacturer’s protocol. At 48 h after transfection, the fluorescence of cells was examined with a Flex Station II microplate reader (Molecular Devices, USA) at 490/510 nm for excitation and emission, respectively. The fluorescence values were corrected by subtracting the autofluorescence of cells not expressing EGFP. All the experiments were biologically repeated three times.

### The detection of mRNA by quantitative real-time PCR

SYBR Green fluorescent quantitative real-time PCR was used to detect the expression of shrimp genes including *STAT*, *JAK*, *Tep1* and *Tep2* at the mRNA level. Shrimp tissues were collected from WSSV-infected shrimp with different treatments at different time points after WSSV infection (0, 24, 36, and 48 h). The total RNAs were extracted from shrimp tissues using RNApure high-purity total RNA rapid extraction kit (Spin-column, BioTeke, Beijing, China) following the manufacturer’s protocol. RNA quality was assessed by electrophoresis on 1.0% agarose gel and the total RNA concentration was determined by measuring the absorbance at 260 nm on a spectrophotometer. The first strand cDNA synthesis was obtained using the PrimeScript^®^ 1st Strand cDNA Synthesis Kit (Takara, Dalian, China) with the Oligo dT Primer. Shrimp β-actin was used as a control. The gene-specific primers (ie1, 5′-TGGCACAACAACAGACCCTA-3′ and 5′-CTTTCCTTGCCGTACGAGAC-3′; STAT, 5′-TGGCAATCAGGAGCCTCATG-3′ and 5′-GGTGTCAAGCATATCTG CAATCTGT-3′; JAK, 5′-CAGGACGAACATCTACTCCAAAT-3′ and 5′-CTCTT GCAGTTCCTTGACAGTTA-3′; Tep1, 5′-CGGCCAAG GTGCGAAATGT-3′ and 5′-AGGTGGAGGGCGTAGGTAGTGAT-3′; Tep2, 5′-A CCCTCGGGACCTGAGG GCCATG-3′ and 5′-CCAGAGTCCGCCGCCGTGAAGA-3′; actin, 5′-CAGCCTTC CTTCCTGGGTATGG-3′ and 5′-GAGGGAGCGAGGGCAGTGATT-3′) were used. Quantitative real-time PCR was performed according to the manufacturer′s instructions in the 2 × SYBR Premix Ex Taq Kit (Takara, Japan) with a real-time thermal cycler (Bio-Rad, Hercules, CA, USA). PCR was conducted at 95 °C for 3 min, followed by 40 cycles of 95 °C for 15 s, and 60 °C for 30 s. Data were quantified by the 2^–△△CT^ method (22) and were subjected to statistical analysis.

### Synthesis of siRNAs and RNAi assay in shrimp *in vivo*

Based on the sequences of shrimp genes including *STAT*, *JAK*, *Tep1*and *Tep2*, siRNAs were separately synthesized according to the design rule for siRNA. The siRNAs used were STAT-siRNA (5′-CCAGUAAAGCCUUCGCCAU-3′), JAK-siRNA (5′-CCATGCCG ATGGCCTATAA-3′), Tep1-siRNA (5′-GGAAGCCA AGTACCTGGAA-3′) and Tep2-siRNA (5′-CCATATAGCCACAGCAACT-3′). The sequences of siRNAs were randomly scrambled to generate the control siRNAs (STAT-siRNA-scrambled, 5′-CU CAAGCGUUAACCACUCC-3′; JAK-siRNA-scrambled, 5′-CCCCGTACCAGATT TAAGG-3′; Tep1-siRNA-scrambled, 5′-CCTACTAAGAGGAGGCAGA-3′; Tep2-siRNA-scrambled, 5′-CTTAACACTAAACACGCGC-3′). The formation of double-stranded RNAs was monitored by determining the size in agarose gel electrophoresis.

The RNA interference (RNAi) assay was conducted in shrimp by the injection of an siRNA into the lateral area of the fourth abdominal segment at 30 μg/shrimp using a 1-ml sterile syringe. The siRNA (15 μg) and WSSV (10^5 ^copies/ml) were co-injected into virus-free shrimp at a volume of 100 μl per shrimp. At 16 h after the co-injection, the siRNA (15 μg) (100 μl/shrimp) was injected into the same shrimp. At the same time, the siRNAs-scrambled (15 μg) (100 μl/shrimp) were co-injected into virus-free shrimp. At 16 h after the co-injection, siRNAs-scrambled (15 μg) (100 μl/shrimp) were injected into the same shrimp. WSSV (10^5 ^copies/ml) (100 μl/shrimp) alone was included in the injections as a positive control. As a negative control, phosphate-buffered saline (PBS) (0.1M, pH7.4) was used in the injections instead of the siRNAs. For each treatment, 20 shrimps were used. The assays were biologically repeated three times.

### Statistical analysis

The data from three independent experiments were analyzed by one-way analysis of variance (ANOVA) to calculate the means and standard deviations (SD) of the triplicate assays^36^.

## Additional Information

**How to cite this article**: Ren, Q. *et al.* A white spot syndrome virus microRNA promotes the virus infection by targeting the host STAT. *Sci. Rep.* **5**, 18384; doi: 10.1038/srep18384 (2015).
